# Relationships between Wheat Development, Soil Properties, and Rhizosphere Mycobiota

**DOI:** 10.3390/microorganisms12081516

**Published:** 2024-07-24

**Authors:** Hang Jiang, Liguo Ma, Peixin Gao, Yueli Zhang, Bo Zhang, Guoping Ma, Kai Qi, Junshan Qi

**Affiliations:** Shandong Key Laboratory for Green Prevention and Control of Agricultural Pests, Institute of Plant Protection, Shandong Academy of Agricultural Sciences, Jinan 250100, China; jhfor724@163.com (H.J.); maliguo809@163.com (L.M.); peixingao123@163.com (P.G.); yueligaoxing@163.com (Y.Z.); zbo8341@163.com (B.Z.); maguopingapple@163.com (G.M.)

**Keywords:** saline–alkali soil, Fusarium crown rot, rhizosphere soil, pH, organic carbon, mineral element, rhizosphere microbial community

## Abstract

Wheat is a vital global food crop, yet it faces challenges in saline–alkali soils where Fusarium crown rot significantly impacts growth. Variations in wheat growth across regions are often attributed to uneven terrain. To explore these disparities, we examined well-growing and poorly growing wheat samples and their rhizosphere soils. Measurements included wheat height, root length, fresh weight, and Fusarium crown rot severity. Well-growing wheat exhibited greater height, root length, and fresh weight, with a lower Fusarium crown rot disease index compared to poorly growing wheat. Analysis of rhizosphere soil revealed higher alkalinity; lower nutrient levels; and elevated Na, K, and Ca levels in poorly growing wheat compared to well-growing wheat. High-throughput sequencing identified a higher proportion of unique operational taxonomic units (OTUs) in poorly growing wheat, suggesting selection for distinct fungal species under stress. FUNGuild analysis indicated a higher prevalence of pathogenic microbial communities in poorly growing wheat rhizosphere soil. This study underscores how uneven terrains in saline–alkali soils affect pH, nutrient dynamics, mineral content, wheat health, and rhizosphere fungal community structure.

## 1. Introduction

Wheat is one of the most important food crops worldwide and is vital for human sustenance and economic development [1]. However, wheat production faces numerous disease challenges, including Fusarium crown rot, Fusarium head blight, wheat stripe rust, powdery mildew, and wheat sharp eyespot [2,3,4]. In China, Fusarium crown rot has become one of the most severe soil-borne diseases affecting wheat [5,6,7]. This disease primarily affects the roots and basal stems of the wheat plant and can result in the entire plant’s death in severe cases. Heavily infected fields can suffer yield losses of up to 30% or more. In China, the predominant pathogen causing Fusarium crown rot is *Fusarium pseudograminearum* [8,9,10]. This pathogen also infects wheat heads and maize roots, leading to Fusarium head blight and maize seedling blight in natural settings [11,12,13]. Besides yield reduction and economic losses, the pathogen produces toxins such as nivalenol (NIV) and deoxynivalenol (DON), which can cause nausea and vomiting if ingested, posing significant health risks to humans and animals [14,15]. The incidence of Fusarium crown rot has been increasing due to the lack of resistant varieties, warming climate, straw returning to fields, and the emergence of fungicide-resistant strains [16,17,18].

China has extensive and widely distributed saline–alkali soils [19], mainly located in Northwest, Northeast, and North China and coastal regions. These saline–alkali conditions negatively impact wheat growth, yield, and quality [20,21]. High salt content, elevated pH levels, and poor water permeability in saline–alkali soils hinder water absorption by wheat roots, impact physiological activities, and impede the uptake of essential nutrients like K, Ca, and Mg, leading to nutrient imbalances and stunted growth [22]. Consequently, these conditions weaken wheat’s disease resistance, making it more susceptible to pathogens and increasing the incidence of diseases like Fusarium crown rot.

Soil properties such as nitrogen content, organic carbon, and rhizosphere microorganisms significantly influence wheat’s growth, development, and health [23,24]. Nitrogen plays a vital role in photosynthesis and metabolism. Sufficient nitrogen supply enhances leaf growth and boosts the efficiency of photosynthesis in wheat [25]. Soil organic carbon provides continuous nutrients and helps improve soil structure, enhancing aggregation and buffering capacity [26]. This makes the soil more resilient to fluctuations in pH and salinity, thereby promoting healthy wheat growth. Rhizosphere microorganisms, which are primarily beneficial, aid in nutrient availability, though some pathogenic microorganisms can negatively affect wheat’s health [27]. Managing these soil properties is essential for ensuring robust and healthy wheat cultivation.

In the Huanghuaihai region, wheat frequently faces drought conditions [28]. The common irrigation method used in this area is flood irrigation. Uneven land surfaces created during farming lead to variations in salinity and alkalinity when flood irrigation is applied to saline–alkali soils. These differences result in varying wheat growth and development across uneven regions of saline–alkali fields. Field surveys conducted in this study revealed significant disparities in wheat growth within the same saline–alkali fields. Statistical analysis showed notable variations in plant height, root length, and fresh weight of wheat in different areas. We propose a hypothesis that the rhizosphere soil properties and rhizosphere microorganisms may differ between wheat plants with good growth and those with poor growth. To validate our hypothesis, we collected rhizosphere soil samples from both well-growing and poorly growing wheat regions and analyzed their pH, total nitrogen content, organic carbon content, and mineral element content. Additionally, we conducted fungal amplicon sequencing of the rhizosphere soil. Our aim is to provide a comprehensive understanding of how soil characteristics and microbial diversity impact wheat growth and health. This research will offer valuable insights to support the development of more effective and sustainable agricultural practices.

## 2. Materials and Methods

### 2.1. Collection of Wheat Plants and Soil Samples

The samples for this study were collected in just a single field in Wulimiao Village, Xushang Street, Shanghe County, Jinan City, Shandong Province, in April 2022. Wheat (variety Jimai 22) was sown in November 2021. This wheat field is located in a saline–alkali area where white salt particles are visibly observed on the soil surface at the field margins. The field has also experienced severe Fusarium crown rot for consecutive years. Due to insufficient rainfall during the wheat growing season in this region, artificial irrigation is necessary. In April 2022, wheat was in the jointing stage. A field survey was conducted in this area, and it was observed that wheat plants showed significant growth differences across different regions. We randomly selected three sampling points in both the well-growing and poorly growing areas of the field. At each sampling point, wheat plants and the soil attached to their roots were collected to gather rhizosphere soil and investigate the occurrence of Fusarium crown rot. The method for collecting rhizosphere soil samples was as follows: first, the loosely attached soil was shaken off the wheat roots, and then, approximately 1 mm of soil covering the roots was collected using a sterile brush [29]. After removing any plant root debris, the collected soil samples were stored at −80 °C.

### 2.2. Investigation of Wheat Disease Incidence and Growth Characteristics

At each sampling point, 20 wheat plants were randomly selected to evaluate the disease index, root length, plant height, and fresh weight of the wheat. The severity of Fusarium crown rot was assessed according to the following criteria: Grade 0 = no symptoms. Grade 1 = lesions on the root or coleoptile. Grade 3 = lesions on the first leaf sheath. Grade 5 = lesions extend to the second leaf sheath. Grade 7 = lesions extend to the third leaf sheath. Grade 9 = lesions extend beyond the third leaf sheath, or the plant shows yellowing and wilting. Based on these evaluation results, the disease index was then calculated. The method for calculating the disease index is as follows:

$$\mathrm{Disease}\;\mathrm{index}=\frac{\sum \left(\mathrm{Disease}\;\mathrm{Rate}\times \mathrm{Number}\;\mathrm{of}\;\mathrm{Plants}\;\mathrm{with}\;\mathrm{this}\;\mathrm{Rate}\right)}{\mathrm{Total}\;\mathrm{Number}\;\mathrm{of}\;\mathrm{Plants}\times \mathrm{Maximum}\;\mathrm{Value}\;\mathrm{of}\;\mathrm{Disease}\;\mathrm{Scale}}\times 100$$

### 2.3. Measurement of Rhizosphere Soil pH

This study measured the pH value of rhizosphere soil using the electrode method. Briefly, 10 g of rhizosphere soil was weighed and ground evenly into a powder. The powder was placed in a clean beaker, and 25 mL of deionized water was added in a soil-to-water ratio of 1:2.5. The mixture was stirred thoroughly for 10 min to ensure complete contact between the soil and water. Then, the suspension was allowed to settle for 30 min. During the standing period, the pH meter was calibrated with standard pH buffer solutions. The electrode was immersed into the soil suspension, ensuring that it was fully in contact with the liquid. The stable reading was read on the pH meter, and the measured pH value was recorded. To ensure accuracy, three repeated measurements were performed for each soil sample, and the average value was calculated.

### 2.4. Measurement of Total Nitrogen, Organic Carbon, and Mineral Element Content in Rhizosphere Soil

The contents of total nitrogen, organic carbon, and mineral elements were measured by SanShu Biotechnology Co., Ltd. (Nantong, China). Determination of total nitrogen content: Samples were digested with sulfuric acid and hydrogen peroxide, oxidizing and decomposing organic matter to convert organic nitrogen in the samples into inorganic ammonium salts. The digestate was then alkalized, and the ammonia was distilled using the SKD-1000 automatic Kjeldahl nitrogen analyzer manufactured by Shanghai Peiou Analytical Instrument Co., Ltd. (Shanghai, China). The distilled ammonia was absorbed by boric acid, and its content was determined by titration using a standard acid solution. Determination of organic carbon content: Under heating conditions, an excess of potassium dichromate–sulfuric acid solution was used to oxidize the soil organic carbon. The excess potassium dichromate was titrated with a standard ammonium ferrous sulfate solution. The difference in potassium dichromate consumption between the sample and a blank was used to calculate the organic carbon content. Determination of mineral element content: The content of mineral elements was determined using the Avio 200 inductively coupled plasma optical emission spectrometer (ICP-OES) manufactured by PerkinElmer, Inc. (Hopkinton, MA, USA). The concentration of each mineral element in the sample was calculated based on the mathematical relationship between the ion concentrations and their peak areas derived from standard solutions of varying concentrations.

### 2.5. ITS Amplicon Sequencing of Rhizosphere Soil

Total genomic DNA was extracted from samples using the CTAB method. The ITS gene V5 region was amplified with specific primers ITS5-1737F/ITS2-2043R (5′-GGAAGTAAAAGTCGTAACAAGG-3′/5′-GCTGCGTTCTTCATCGATGC-3′). Sequencing libraries were prepared using the TruSeq DNA PCR-Free Sample Preparation Kit (Illumina, San Diego, CA, USA) following the manufacturer’s instructions. Library quality was evaluated using the Qubit 2.0 Fluorometer and Agilent Bioanalyzer 2100 system manufactured by Agilent Technologies (Santa Clara, CA, USA). Subsequently, the libraries were sequenced on an Illumina NovaSeq platform to generate 250 bp paired-end reads at the Novogene Bioinformatics Technology Co., Ltd. (Beijing, China). Paired-end reads were merged using Flash 1.2.7 [30]. Raw tags underwent quality filtering under specific conditions to obtain high-quality clean tags [31], following the quality control process of QIIME 1.9.1 [32]. The tags were then compared with the Unite Database (https://unite.ut.ee/, accessed on 10 October 2022) [33] using vsearch (https://github.com/torognes/vsearch/, accessed on 10 October 2022) [34].

### 2.6. Sequencing Data Analysis

Sequencing data analysis was performed on the Novomagic platform (https://magic.novogene.com, accessed on 10 October 2022). Sequence analysis utilized Uparse 7.0.1001 [35]. Sequences with ≥97% similarity were grouped into the same OTUs (Operational Taxonomic Units). Taxonomic information for each representative sequence was annotated using the Unite Database (https://unite.ut.ee/, accessed on 10 October 2022) [33] based on the BLAST algorithm. Multiple sequence alignment was conducted using MUSCLE 3.8.31 [36] to explore the phylogenetic relationships among different OTUs and to examine the differences in dominant species across samples. Alpha diversity metrics, including OTU richness, Shannon, and Simpson indices, were calculated to assess species diversity complexity within samples. Principal Coordinate Analysis (PCoA) was performed to extract principal coordinates and visualize patterns from the complex, multidimensional data. PCoA results were displayed using the ade4 and ggplot2 packages in R 2.15.3. Additionally, the package vegan was employed in R 2.15.3 to conduct Mantel tests and Spearman correlation analyses, assessing the relationships between environmental factors and microbial community composition and abundance. The DESeq2 package in R 4.3.1 was used to compute the log_2_FC (fold changes) of OTUs based on the negative binomial distribution. OTUs were selected only when the adjusted *p*-value (*p*_adj_) was less than 0.05, and the absolute log_2_FC was greater than 1. For all comparisons and statistical tests, *p*-values were adjusted using the Benjamini–Hochberg method, with the significance threshold set at alpha = 0.05 [37].

## 3. Results

### 3.1. Variation in Wheat Growth in Saline–Alkali Soil

In April 2022, severe Fusarium crown rot was observed in Shanghe County, Jinan City, Shandong Province. The affected fields are located on saline–alkali land, as evidenced by the presence of white granules on the soil surface at the field margins. During our investigation of Fusarium crown rot, we noted that wheat growth varied significantly within the same field. In some areas, wheat exhibited robust growth, while in others, growth was notably poorer (Supplementary Figure S1). Upon closer inspection, the wheat with poorer growth was predominantly found in low-lying areas of the field.

### 3.2. Disease Index and Wheat Growth Characteristics

**Figure 1:**
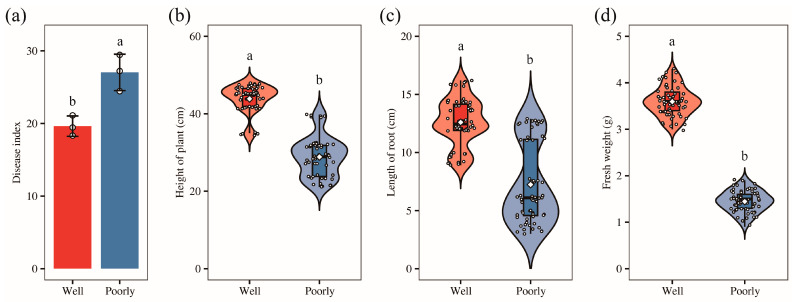
The disease index (**a**), plant height (**b**), root length (**c**), and fresh weight (**d**) of the well-growing (Well) and poorly growing (Poorly) wheat. Different letters indicate significant differences as determined by Duncan’s pairwise comparison (*p* < 0.05).

To investigate the underlying causes of the observed variations in wheat growth, we collected samples from both well-growing and poorly growing areas within the field. Most of these wheat plants were infected with Fusarium crown rot. Our survey revealed that the disease index for Fusarium crown rot was 19.63 in the well-growing wheat and 27.04 in the poorly growing wheat. This indicates a significantly higher disease index in the poorly growing wheat compared to the well-growing wheat (*p* < 0.05) (Figure 1a). Further measurements showed that plant height, root length, and fresh weight were significantly greater in wheat from the well-growing areas than those from the poorly growing areas (*p* < 0.05) (Figure 1b–d). These findings align with our field observations.

### 3.3. Determination of Soil pH

**Figure 2:**
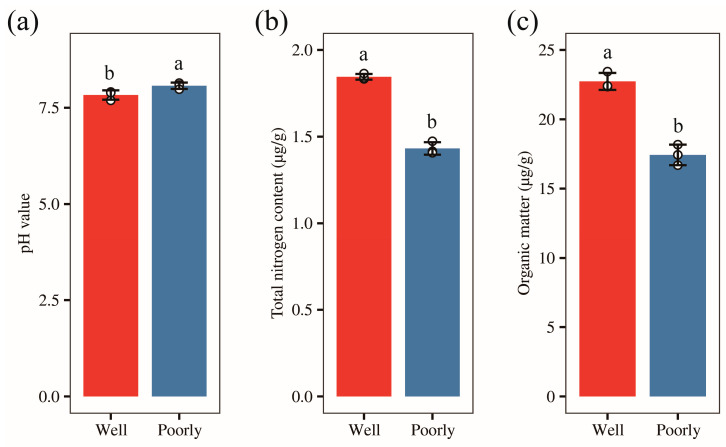
The pH value (**a**), total nitrogen content (**b**), and organic carbon content (**c**) of rhizosphere soil in well-growing (Well) and poorly growing (Poorly) wheat. Different letters indicate significant differences as determined by Duncan’s pairwise comparison (*p* < 0.05).

We collected rhizosphere soil from both well-growing and poorly growing wheat. To clarify the salinity and alkalinity levels of the rhizosphere soil in the areas with different wheat growth conditions, we measured the pH values of the rhizosphere soil in these two areas, respectively. The results showed that the rhizosphere soil in both areas was alkaline, with pH values of 7.83 and 8.07 in well-growing wheat and poorly growing wheat, respectively (Figure 2a). The soil pH in areas with poorly growing wheat was significantly higher than in areas with well-growing wheat (*p* < 0.05). This suggests that the rhizosphere soil of poorly growing wheat is more alkaline compared to that of well-growing wheat.

### 3.4. Analysis of Total Nitrogen and Organic Carbon Content in Wheat Rhizosphere Soil

To further understand the nutrient status in wheat rhizosphere soil, we measured the total nitrogen and organic carbon content of the rhizosphere soil. Our results revealed that the total nitrogen content in the rhizosphere soil of well-growing wheat was 1.85 g/kg, compared to 1.43 g/kg in poorly growing wheat. Similarly, the organic carbon content was 13.19 g/kg for well-growing wheat and 10.11 g/kg for poorly growing wheat. Both the total nitrogen and organic carbon levels were significantly higher in the rhizosphere soil of well-growing wheat than in poorly growing wheat (*p* < 0.05) (Figure 2b,c). These results indicate that the rhizosphere soil of well-growing wheat is more nutrient-rich compared to that of poorly growing wheat.

### 3.5. Mineral Elements Content in Wheat Rhizosphere Soil

**Figure 3:**
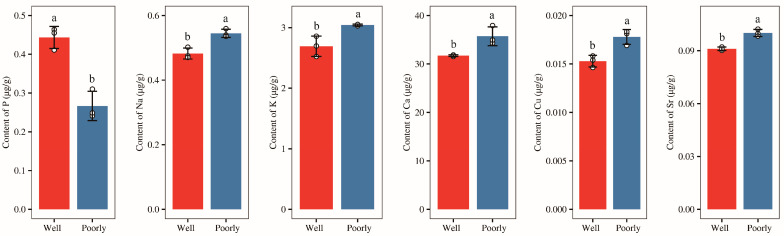
The contents of the mineral elements P, Na, K, Ca, Cu, and Sr in the rhizosphere soil of well-growing (Well) and poorly growing (Poorly) wheat. Different letters indicate significant differences as determined by Duncan’s pairwise comparison (*p* < 0.05).

Afterward, we measured the content of mineral elements (P, K, Na, Mg, Al, Fe, Ca, B, Ti, V, Cr, Mn, Li, Ni, Cu, Zn, Sr, and Ba) in the rhizosphere soil of wheat and found that the levels of minerals Mg, Al, Fe, B, Ti, V, Cr, Mn, Li, Ni, Zn, and Ba in rhizosphere of well-growing wheat had no significantly difference with those in poorly growing wheat (Supplementary Figure S2). However, the level of mineral P was significantly higher in the rhizosphere soil of well-growing wheat compared to poorly growing wheat (*p* < 0.05) (Figure 3). Conversely, the levels of minerals K, Na, Ca, Cu, and Sr were significantly lower in the rhizosphere soil of well-growing wheat than in poorly growing wheat (*p* < 0.05) (Figure 3). Additionally, no detectable levels of Be, As, Se, Mo, Cd, Sb, or Tl were found in the rhizosphere soil of either well-growing or poorly growing wheat.

### 3.6. Analysis of Wheat Rhizosphere Microbial Communities

To further elucidate the differences in the rhizosphere soil mycobiota between well-growing and poorly growing wheat, we conducted ITS amplicon sequencing on the rhizosphere soil of both wheat types.

#### 3.6.1. Alpha Diversity Analysis of Microbial Communities

We analyzed the alpha diversity of the fungal microbiome in the rhizosphere soils. The results indicated no significant differences in OTU richness, Shannon, Simpson, and phylogenetic diversity indices between the rhizosphere soils of well-growing and poorly growing wheat (Table 1). Additionally, the rarefaction curves for rhizosphere microbes showed no significant differences in the structure and diversity of microbial communities between the two groups (Supplementary Figure S3). This suggests that the richness and phylogenetic diversity of the rhizosphere fungal communities in well-growing and poorly growing wheat are fundamentally similar.

#### 3.6.2. Beta Diversity Analysis of Microbial Communities

**Figure 4:**
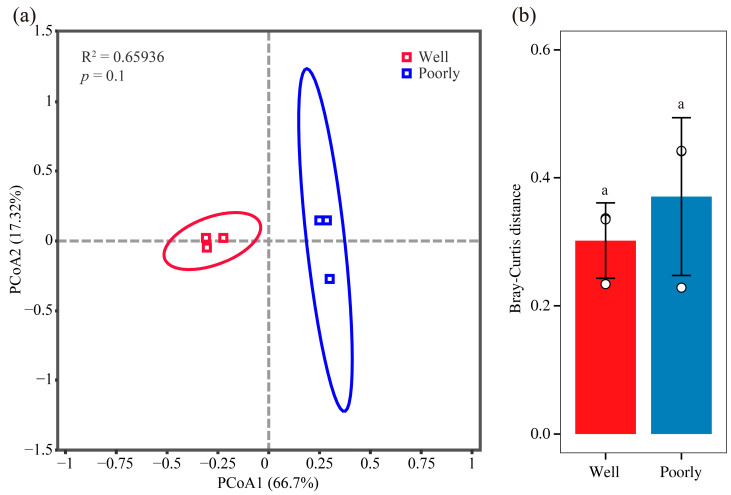
Beta diversity analysis of fungal communities. PCoA analysis (**a**) and Bray–Curtis distance (**b**) of rhizosphere soil in well-growing (Well) and poorly growing (Poorly) wheat. Different letters indicate significant differences as determined by Duncan’s pairwise comparison (*p* < 0.05).

We visualized the structure of the rhizosphere fungal communities in well-growing and poorly growing wheat soil using PCoA. The PCoA plot revealed that the soil samples from well-growing and poorly growing wheat formed distinct clusters in the ordination space (Figure 4a). However, the Bray–Curtis distance analysis indicated that there was no significant difference in the beta diversity of the microbial communities between the well-growing and poorly growing wheat groups (Figure 4b). This suggests that while the microbial community composition appears to cluster separately for the two wheat growth conditions, the overall diversity and structure of these communities did not significantly differ between well-growing and poorly growing wheat.

#### 3.6.3. Microbial Community Composition

**Figure 5:**
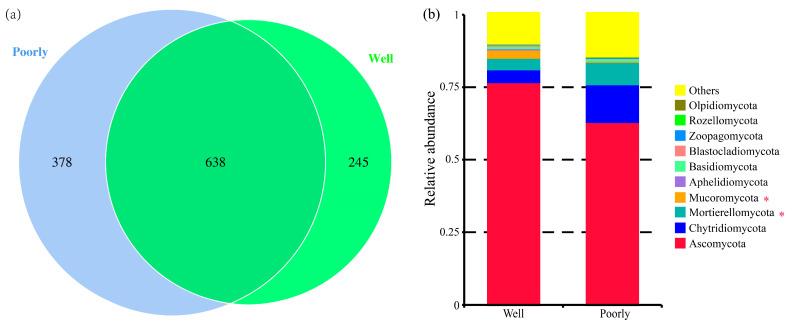
Venn diagram for the number of fungal OTUs (**a**) and relative abundance of fungal communities at the phylum level (**b**) in the rhizosphere soil of well-growing (Well) and poorly growing (Poorly) wheat. * indicates significant differences as determined by Duncan’s pairwise comparison (*p* < 0.05).

A total of 1261 fungal OTUs were identified across all samples (Supplementary Table S1). Specifically, 883 OTUs were identified in the rhizosphere soil of well-growing wheat, while 1016 OTUs were identified in the rhizosphere soil of poorly growing wheat (Figure 5a). Although many OTUs were common to both conditions, the proportion of unique OTUs was higher in the rhizosphere soil of poorly growing wheat (37.20%) compared to well-growing wheat (27.75%). This indicates that the soil environment of poorly growing wheat may exert a more diverse and potentially stimulating effect on the fungal community.

The analysis of the relative abundance of fungi at the phylum level indicated differences in the fungal community composition between the rhizosphere soils of well-growing and poorly growing wheat. Ascomycota was the dominant phylum, accounting for 42.31% to 78.22% of the relative abundance. Notably, the relative abundance of Mucoromycota was significantly higher in the rhizosphere soil of well-growing wheat compared to poorly growing wheat, whereas Mortierellomycota was significantly more abundant in the rhizosphere soil of poorly growing wheat than in well-growing wheat (*p* < 0.05) (Figure 5b).

#### 3.6.4. Differential Analysis of Fungal OTUs in Wheat Rhizosphere Soil

**Figure 6:**
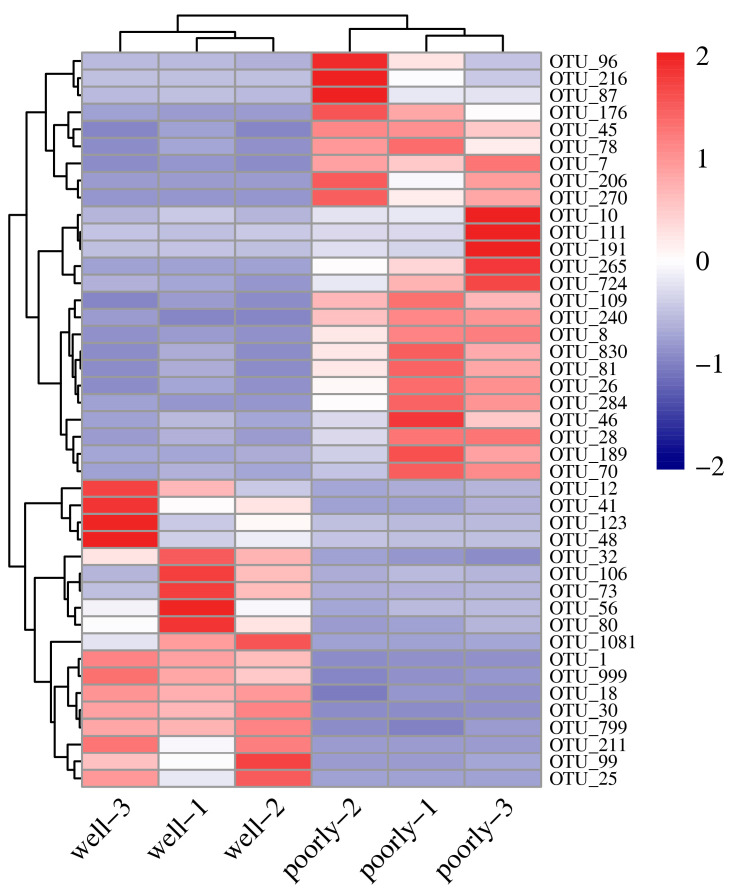
Heatmap showing differentially abundant fungal OTUs identified in the rhizosphere soil from well-growing (Well) and poorly growing (Poorly) wheat. OTUs with an absolute log_2_FC > 1 and *p*_adj_ < 0.05 were considered differentially abundant.

According to DESeq2 analysis, 18 OTUs were significantly reduced in the microbial community of poorly growing wheat’s rhizosphere soil compared to that of well-growing wheat (log_2_FC < −1, *p*_adj_ < 0.05). These OTUs span various phyla, including Aphelidiomycota, Ascomycota, Basidiomycota, Chytridiomycota, Mortierellomycota, Mucoromycota, and Rozellomycota, with Ascomycota comprising 44.44% of the reduced OTUs (Figure 6 and Supplementary Table S2). Conversely, 25 OTUs were significantly increased in the microbial community of poorly growing wheat’s rhizosphere soil compared to well-growing wheat (log_2_FC > 1, *p*_adj_ < 0.05). These OTUs belong to Ascomycota, Basidiomycota, Chytridiomycota, and Zoopagomycota, with Ascomycota accounting for 72% of the increased OTUs. Therefore, Ascomycota may have a closer association with Fusarium crown rot, a disease affecting poorly growing wheat (Figure 6 and Supplementary Table S2).

#### 3.6.5. FUNGuild Analysis of Fungal Communities

**Figure 7:**
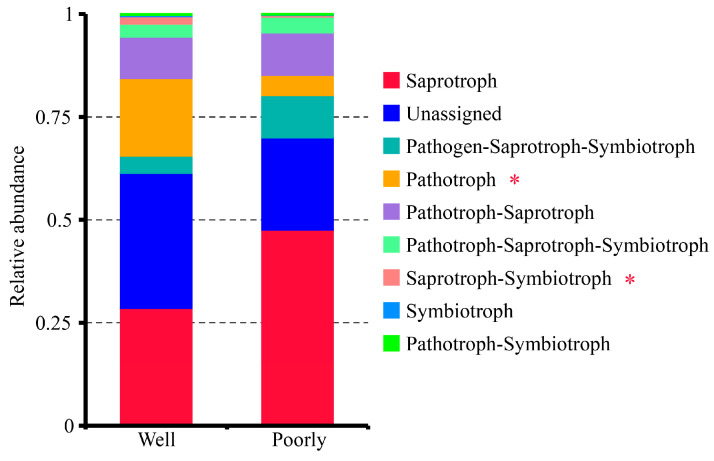
Relative abundance of trophic modes assigned by FUNGuild for fungal communities in the rhizosphere soil of well-growing (Well) and poorly growing (Poorly) wheat. * indicates significant differences as determined by Duncan’s pairwise comparison (*p* < 0.05).

The FUNGuild analysis classified the fungal communities in the rhizosphere soil into seven trophic modes: Pathogen–Saprotroph–Symbiotroph, Pathotroph, Pathotroph–Saprotroph, Pathotroph–Saprotroph–Symbiotroph, Pathotroph–Symbiotroph, Saprotroph, Saprotroph–Symbiotroph, and Symbiotroph (Figure 7). Among these, the Saprotroph mode was the most dominant. Notably, the rhizosphere soil of poorly growing wheat had significantly higher proportions of the Pathotroph and Saprotroph–Symbiotroph groups compared to well-growing wheat (*p* < 0.05) (Figure 7). This suggests a higher presence of pathogenic fungi in the microbial communities of the rhizosphere soil associated with poorly growing wheat, potentially contributing to its poor growth.

### 3.7. Correlation between Microbial Communities and Environmental Factors

**Figure 8:**
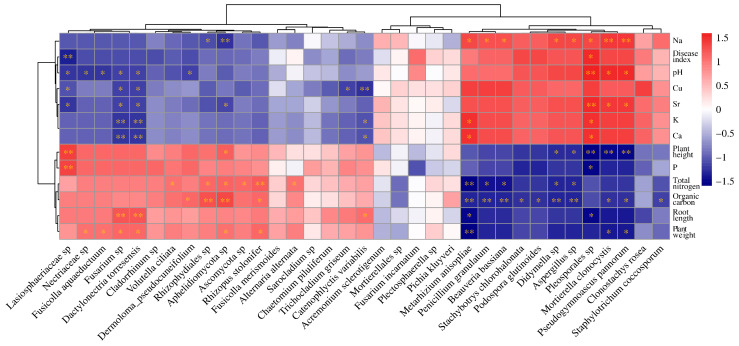
The Spearman correlation heatmap illustrates the relationships between environmental factors and fungal community composition. * indicates *p* < 0.05, and ** indicates *p* < 0.01.

Mantel test analysis revealed significant correlations between the microbial community composition and seven environmental factors, including root length, plant weight, total nitrogen, organic carbon, Na, Ca, and Sr (Supplementary Figure S4). Further, Spearman correlation analysis provided deeper insights into these relationships. It showed that the abundance of pathogenic fungi *Fusarium* spp. and *Dactylonectria torresensis* decreased with increasing levels of pH, Cu, Ca, K, and Sr (Figure 8). This indicates that environments with higher pH and elevated mineral content are less conducive to the growth of these pathogens. Additionally, the abundance of symbiotic fungi *Metarhizium anisopliae* and *Beauveria bassiana* was inversely correlated with nitrogen and organic carbon (Figure 8). This indicates that high-nutrient conditions, characterized by elevated total nitrogen and organic carbon, may be unfavorable for these symbiotic fungi. Overall, total nitrogen and organic carbon showed significant correlations with the abundance of many fungi (Figure 8), emphasizing the critical role of soil nutrient conditions in regulating fungal community composition and growth.

## 4. Discussion

This study highlights the significant impact of soil properties on the development and health of wheat and the composition of rhizosphere soil. By examining the interplay between soil pH, nutrient content, and microbial diversity in areas of varied wheat growth, we can better understand the underlying factors influencing wheat health and productivity in saline–alkali environments. One of the key findings of this study is the higher alkalinity in the rhizosphere soil of poorly growing wheat compared to well-growing wheat. The pH levels measured in the poorly growing areas were significantly higher (more alkaline) than those in the well-growing regions. This increased alkalinity is often attributed to the low-lying topography of these areas, which can lead to the accumulation of salts and reduced drainage. Higher soil alkalinity likely plays a crucial role in impeding nutrient absorption and overall plant health [38]. Previous studies have shown that alkaline soils can restrict the availability of essential nutrients, such as Fe, Mn, and P, which are critical for plant development and metabolic functions [39]. These nutrient limitations may explain the poorer performance of wheat in these high-pH, saline–alkali environments.

Under salt-induced conditions, plants undergo several adaptive responses, such as the regulation of phytohormones, melatonin, ion transport, mitochondrial respiration, and growth and development levels, as well as the activation of reactive oxygen species (ROS) cascades [40,41,42,43]. While these mechanisms are essential for plant adaptation to stress, they can simultaneously compromise the plant’s resistance to pathogens, making them more susceptible to diseases like Fusarium crown rot. Our study highlights a significant difference in mineral content, particularly the elevated levels of K, Na, Ca, Cu, and Sr in the rhizosphere soil of poorly growing wheat. This suggests that salinity and mineral imbalances in these areas may exacerbate the challenges faced by the plants. High concentrations of certain minerals, especially sodium, can cause osmotic stress and ion toxicity, adversely affecting plant growth and development [44,45]. These conditions likely create an environment that is less conducive to the healthy growth of wheat, leading to the observed variations in plant height, root length, and fresh weight.

Interestingly, poorly growing wheat exhibited a higher proportion of unique OTUs in its rhizosphere soil. This suggests that the stressed environment in poorly growing areas might select for a more diverse range of fungal species, potentially including opportunistic pathogens that thrive in saline–alkali conditions. Some inorganic salts have been shown to significantly inhibit various fungal diseases, including wheat stripe rust, wheat powdery mildew, and grape powdery mildew [46]. In our study, the presence of pathogenic fungi such as *Fusarium* spp. and *Dactylonectria torresensis* [47] was found to decrease with higher levels of pH, Cu, Ca, K, and Sr. This inverse relationship suggests that while these conditions might be unfavorable for plants, they may also suppress the growth of certain pathogens. The FUNGuild analysis revealed that poorly growing wheat had significantly higher proportions of Pathotroph and Saprotroph–Symbiotroph groups compared to well-growing wheat. This points to a more pathogenic and decomposer-dominated microbial environment in the poorly growing wheat’s rhizosphere soil. Such conditions could further stress the plants and reduce their growth and yield potential.

These findings underscore the critical importance of managing soil pH and nutrient levels to promote healthy wheat growth, especially in saline–alkali terrains. Enhancing soil organic carbon and nitrogen levels, along with careful management of mineral content, could mitigate the adverse effects of salinity and alkalinity. Practices such as soil amendments [48,49], crop rotation [50], and the use of salt-tolerant wheat varieties [51,52] might be effective strategies for improving wheat productivity in these challenging environments. Maintaining level terrain during cultivation is also crucial to minimize waterlogging and salt accumulation. Moreover, understanding the microbial dynamics in the rhizosphere can guide the development of biocontrol and biofertilization strategies to harness beneficial fungi and suppress pathogens [53,54]. For instance, promoting the growth of symbiotic fungi and reducing the prevalence of pathogenic fungi through targeted soil management practices could enhance wheat resilience and yield in saline–alkali soils.

## 5. Conclusions

In conclusion, this study highlights the significant influence of uneven terrain and saline–alkali conditions on wheat growth and microbial diversity. Compared to the rhizosphere soil of well-growing wheat, the soil of poorly growing wheat exhibited higher pH values and increased levels of Na, K, Ca, Cu, and Sr while showing lower levels of nitrogen, organic carbon, and P. Additionally, the proportion of unique OTUs was notably higher in the rhizosphere soil of poorly growing wheat compared to that of well-growing wheat. Furthermore, the rhizosphere soil of poorly growing wheat contained significantly higher proportions of the Pathotroph group. Our findings offer valuable insights into the complex interactions between soil properties and wheat growth, emphasizing the need for integrated soil and crop management strategies to enhance wheat production in saline–alkali fields.

## Figures and Tables

**Table 1:** Comparison of alpha diversity indices of rhizosphere fungal communities.

Sample Name	OTU Richness	Shannon	Simpson	Phylogenetic Diversity
Well-growing wheat	518.67 ± 34.69 a ^#^	4.82 ± 0.16 a	0.89 ± 0.01 a	173.16 ± 10.42 a
Poorly growing wheat	606.67 ± 56.67 a	5.30 ± 0.18 a	0.93 ± 0.02 a	211.67 ± 22.45 a

^#^ Results show mean ± SE. Different letters indicate significant differences as determined by Duncan’s pairwise comparison (*p* < 0.05).

## Data Availability

The original contributions presented in the study are included in the article/Supplementary Materials, further inquiries can be directed to the corresponding author. The microbiome data generated in this study have been deposited in the NCBI Sequence Read Archive database under the accession code PRJNA1138460.

## Supplementary Materials

The following supporting information can be downloaded at https://www.mdpi.com/article/10.3390/microorganisms12081516/s1. Figure S1: Image depicting wheat in the field with varying growth conditions; Figure S2: Mineral elements contents of rhizosphere soil in well-growing (Well) and poorly growing (Poorly) wheat. Different letters indicate significant differences as determined by Duncan’s pairwise comparison (*p* < 0.05); Figure S3: Rarefaction curves illustrating the relationship between sequence number and OTU number for well-growing and poorly growing rhizosphere soil microbiome samples; Figure S4: Mantel Test results showing the relationship between environmental factors and rhizosphere soil fungal communities. * indicates significant differences as determined by Duncan’s pairwise comparison (*p* < 0.05); Table S1: Identified OTUs in all rhizosphere soil samples from this study; Table S2: Differentially abundant fungal OTUs in the rhizosphere soil of poorly growing wheat compared to that in well-growing wheat rhizosphere soil.
