# Supplementary figures and images for: Relationships between Wheat Development, Soil Properties, and Rhizosphere Mycobiota
Source: Microorganisms. 2024 Jul 24;12(8):1516. doi: 10.3390/microorganisms12081516 (PMC11356171; doi:10.3390/microorganisms12081516)

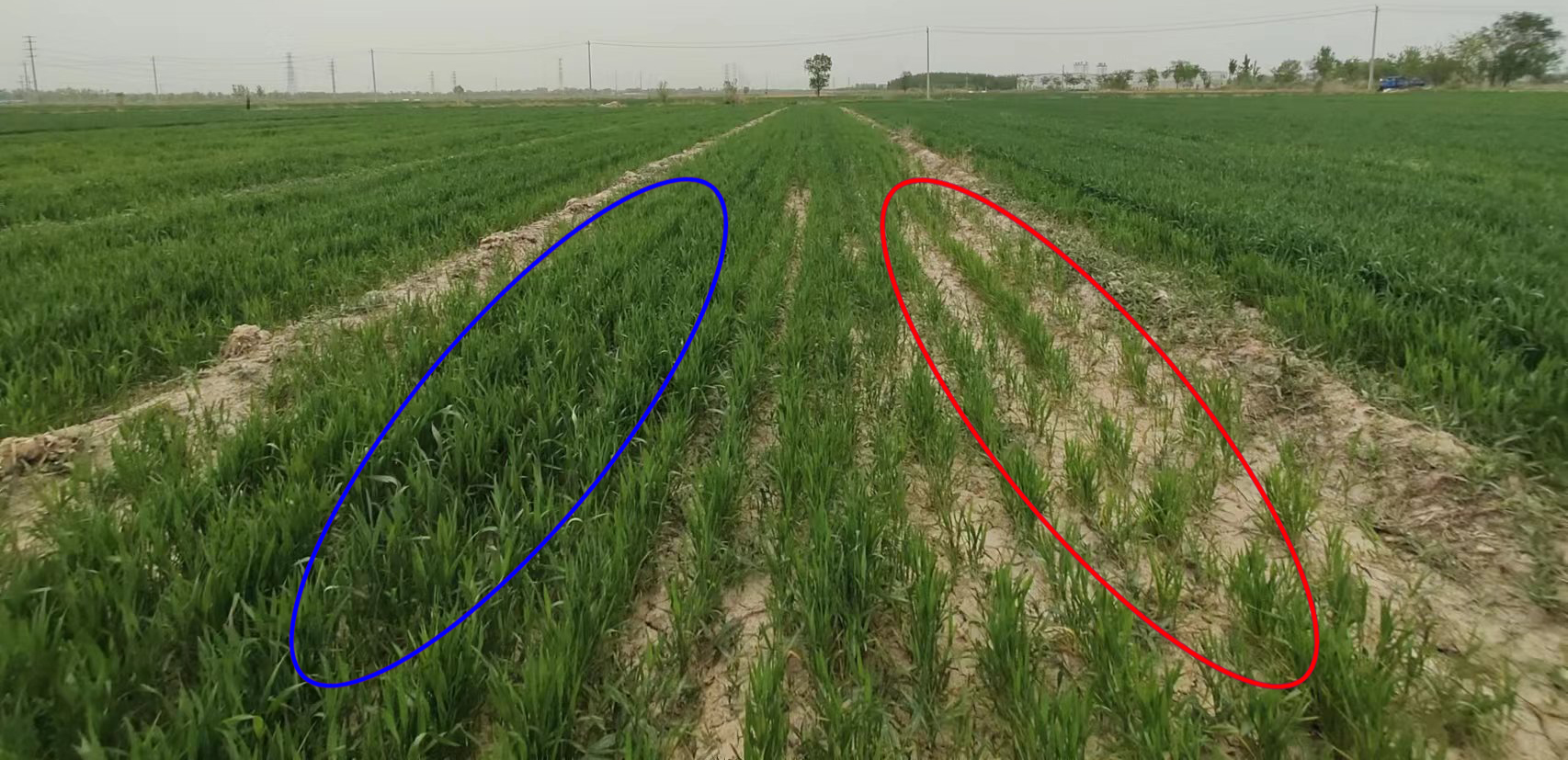

Supplement: Supplementary file 1 [file microorganisms-12-01516-s001.zip › microorganisms-3111124-supplementary/FigureS1.jpg]

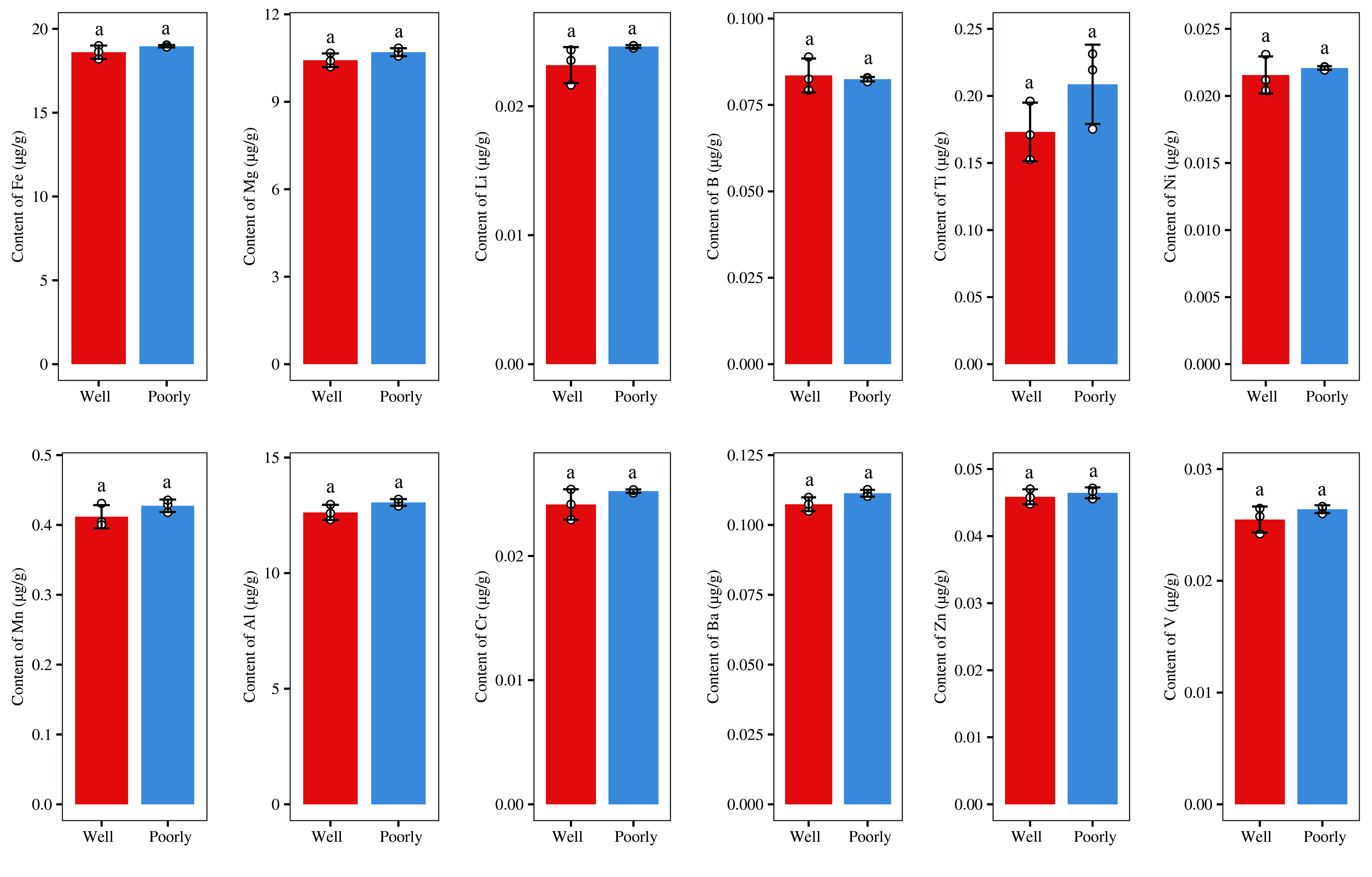

Supplement: Supplementary file 1 [file microorganisms-12-01516-s001.zip › microorganisms-3111124-supplementary/FigureS2-mineralelement.jpg]

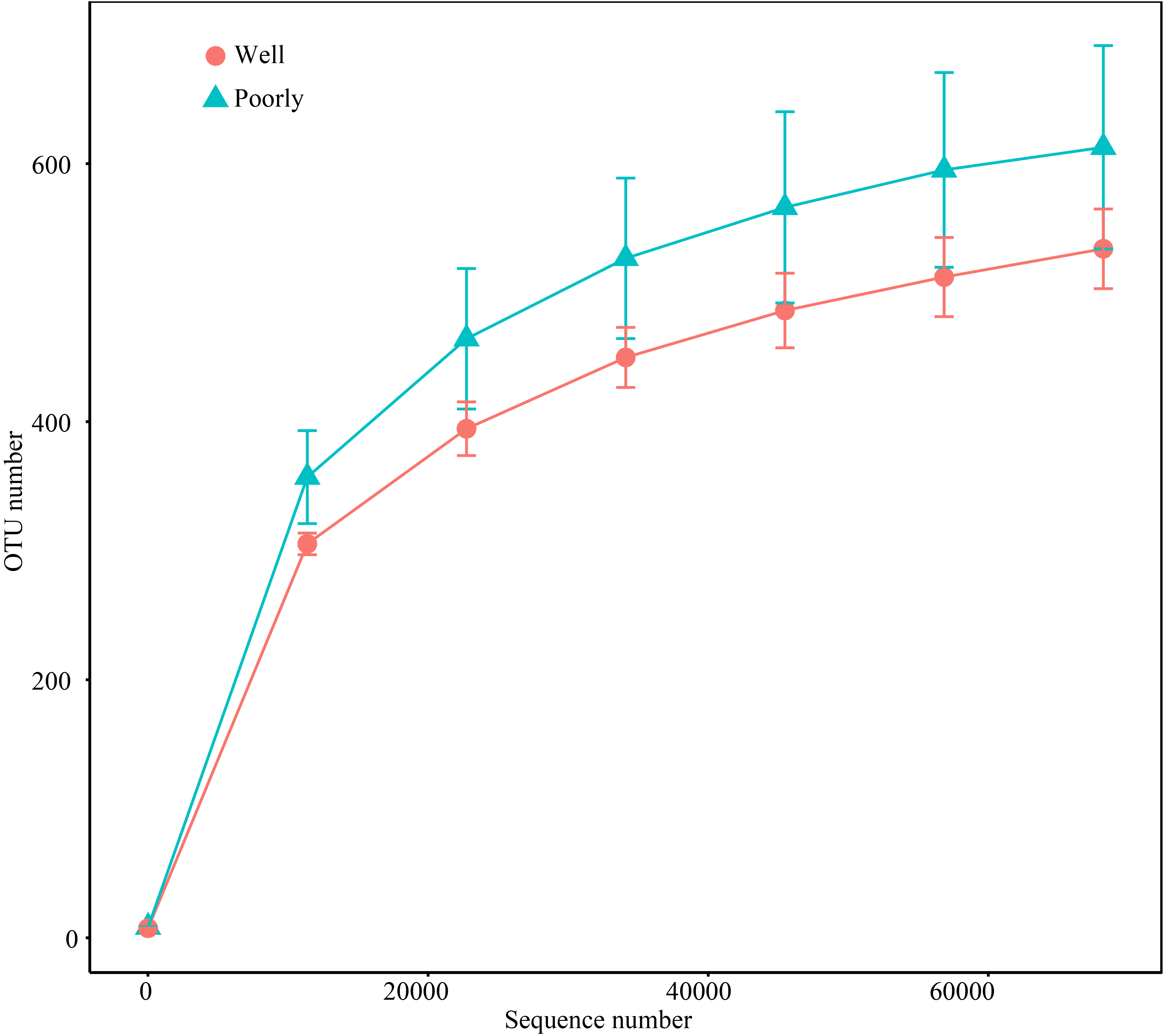

Supplement: Supplementary file 1 [file microorganisms-12-01516-s001.zip › microorganisms-3111124-supplementary/FigureS3-rare otu.jpg]

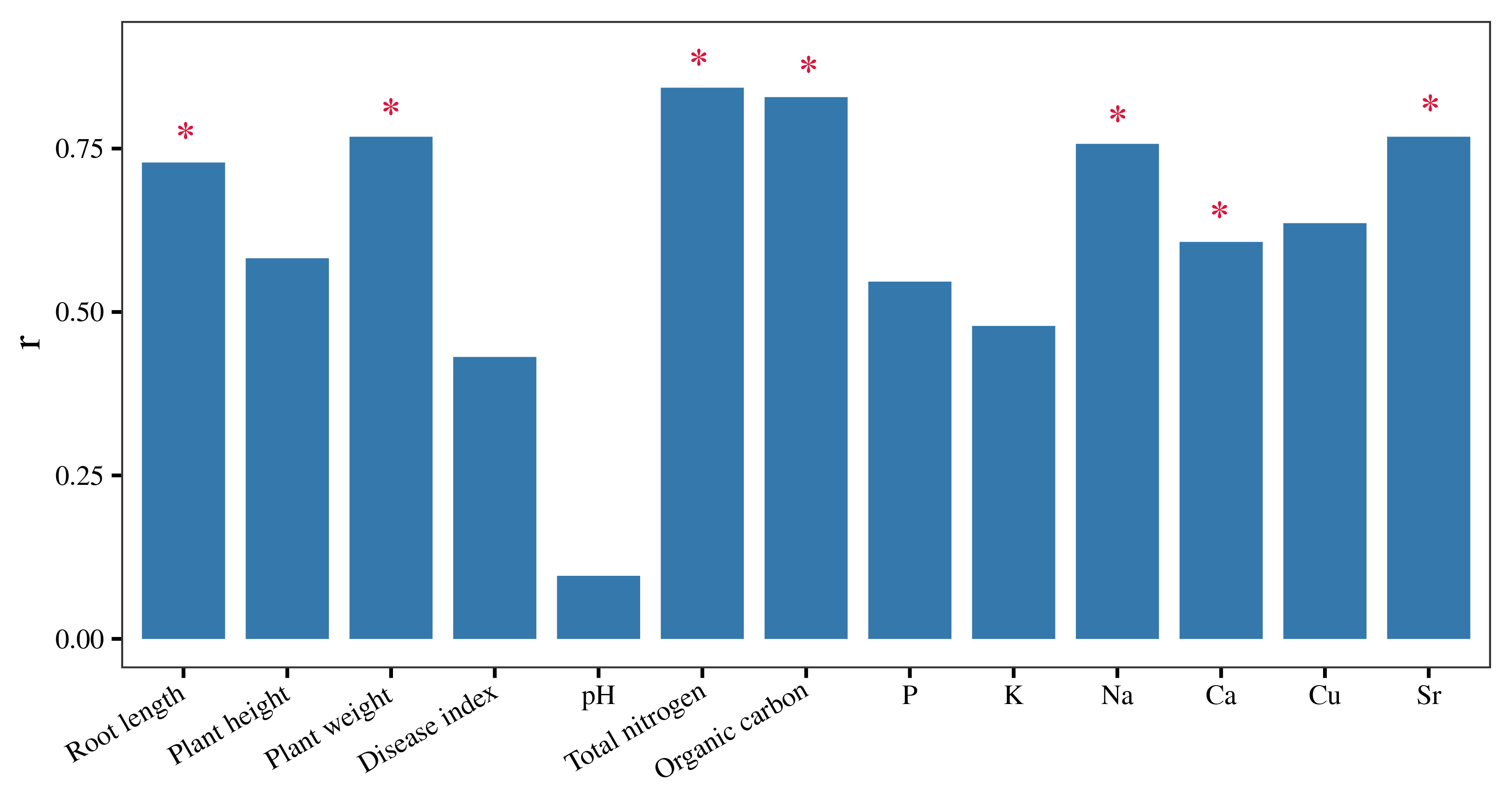

Supplement: Supplementary file 1 [file microorganisms-12-01516-s001.zip › microorganisms-3111124-supplementary/FigureS4-mantaltest.jpg]
